# UV/O_3_ treatment as a surface modification of rice husk towards preparation of novel biocomposites

**DOI:** 10.1371/journal.pone.0197345

**Published:** 2018-05-30

**Authors:** Nishata Royan Rajendran Royan, Abu Bakar Sulong, Nor Yuliana Yuhana, Ruey Shan Chen, Mohd Hafizuddin Ab Ghani, Sahrim Ahmad

**Affiliations:** 1 Faculty of Engineering and Built Environment, Universiti Kebangsaan Malaysia, Bangi, Selangor, Malaysia; 2 School of Applied Physics, Faculty of Science and Technology, Universiti Kebangsaan Malaysia, Bangi, Selangor, Malaysia; Michigan Technological University, UNITED STATES

## Abstract

The use of rice husks (RH) to reinforce polymers in biocomposites are increasing tremendously. However, the incompatibility between the hydrophilic RH fibers and the hydrophobic thermoplastic matrices leads to unsatisfactory biocomposites. Surface modification of the fiber surface was carried out to improve the adhesion between fiber and matrix. In this study, the effect of surface modification of RH via alkali, acid and ultraviolet-ozonolysis (UV/O_3_) treatments on the properties of composites recycled high density polyethylene (rHDPE) composites was investigated. The untreated and treated RH were characterized by Fourier Transform Infrared (FTIR) and Scanning Electron Microscope (SEM). The composites containing 30 wt% of RH (treated and untreated) were then prepared via extrusion and followed by compression molding. As compared to untreated RH, all surface treated RH exhibited rougher surface and showed improved adhesion with rHDPE matrix. Tensile strength of UV/O_3_-treated RH composites showed an optimum result at 18.37 MPa which improved about 5% in comparison to the composites filled with untreated RH. UV/O_3_ treatment promotes shorter processing time and lesser raw material waste during treatment process where this is beneficial for commercialization in the future developments of wood plastic composites (WPCs). Therefore, UV/O_3_ treatment can be served as an alternative new method to modify RH surface in order to improve the adhesion between hydrophilic RH fibre and hydrophobic rHDPE polymer matrix.

## Introduction

Recently, there is an increase of research studies and progresses in the wood plastic composite (WPC) technology. The use of natural fibers to reinforce polymers is known as a well-established practice. Currently, biocomposites have been used in various sectors such as automotive and construction to a great extent. One of a specific class of biocomposites is green composites where a bio-based polymer matrix is reinforced with natural fibers and they are growing tremendously in polymer science [1]. The reasons behind this rapid growth are due to their low costs, biodegradability, environmental friendly, low density, non-hazardous, nonabrasive nature and wide variety of fiber types [2]. Because of the large quantity of plastics wastes, including polyethylene and etc., generated daily, the development of a new value added composite with the use of the consumer or recovered plastics is assumed to have great prominence. The addition of fibers (eg. rice husk (RH)) to waste plastics (eg. recycled high-density polyethylene (rHDPE)) makes the resulting composites viable from both the properties and the environmental points of view, which are lower material cost, lighter weight as well as improved stiffness, acoustic, impact, and heat reform abilities properties compared to products made from plastics alone [3]. RH is known as the agricultural waste crops and was estimated that approximately 20% of RH can be obtained from the total generation of rice during the milling process. Generally, the chemical composition of RH consists of 35% cellulose, 25% hemicellulose, 20% lignin and 17% ash (94% silica), by weight [4].

However, the incompatibility between the hydrophilic natural fibers and the hydrophobic thermoplastic matrices acts as a certain drawback of natural fibres/polymers composites. This has led to undesirable properties for the final composites [5, 6]. In order to improve the adhesion between fibre and matrix, it is necessary to modify the fibre surface by applying some chemical modifications [7]. Chemical surface modification using the alkaline treatment is a well-established method where sodium hydroxide (NaOH) has been used to treat RH cellulosic fibres and so to improve the fibre-polymer matrix interface bonding [8]. The importance of alkaline treatment could be the disruption of hydrogen bonding in the chain structure of natural fiber where this increases the surface roughness. Besides depolymerizing the cellulose component and exposing the short length crystallites, this alkaline treatment also removes certain amount of lignin, wax and oils which covers the external surface of the RH fiber’s cell wall [9]. Meanwhile, acid treatment aids to eliminate inorganic materials, such as carbonate and silica, from the rice husk surface [10]. Similar to alkaline treatment, the removal of the surface impurities have improved the surface roughness of the fibers or particles, thus opening more hydroxyl groups and other reactive functional groups on the surface [11].

There are many research works reported on the types of surface modifications carried out on RH, typically the alkali [12–14] and acid treatment [10, 11, 15, 16]. Hu and Lim reported that there was a significant improvement in the tensile properties of hemp fiber-reinforced polylactic acid (PLA) upon alkali treatment on fiber as compared to those untreated one[13]. Liu et al. evaluated that different composition of alkali treatment increased the tensile strength and modulus of jute fibre composites. The author also reported that alkaline treatment increased the surface roughness, improved the surface impurities, and reduced the diameter of jute fiber [14]. The later treatment has been carried out by Li et al. on flax fibres-reinforced polyethylene biocomposites using acrylic acid, and the results showed that the acrylic acid treatment exhibited higher effectiveness than alkali and silane treatment, in term of tensile properties [17]. Zeynab et al. reported that the use of nitric acid (HNO_3_) treatment on RH surface increased the water absorption property where the material became more porous and created surface hydroxyl group after acid treament[15]. However, very few papers reported on the ozone surface modification method. Ozone (O_3_) treatment will attack the aromatic ring structures which cause the degradation of lignin while hemicellulose and cellulose are not damaged at the same process time [18]. Ozone treatment also can be used to disrupt and agitate the structure of many different lignocellulosic materials, such as bagasse, pine, wheat straw, peanut, poplar sawdust and cotton straw [19]. According to Gassan et al. [20], the ultraviolet ray (UV) treatment of jute fibers caused the higher polarities of the fiber surface and subsequently improved the wettability of fibers and the epoxy composites. The exposure to UV and O_3_ in combination has been used as a surface modification for polymer [21, 22], nanoscale filler/particles such as carbon-based materials [23–26], etc. Yet, this surface modification method is not widely applied on natural fibers [27]. In which this combined treatment (UV/O_3_ treatment) was used as dry surface modification method for RH filler in this study.

Despite of these studies, the studies on the comparison of surface modification on wet and dry oxidation treatments are still limited. The objective of this study was aimed to evaluate the effect of surface modified RH on the mechanical properties of biocomposites. Three types of surface modification were applied in this study which are the alkali, acid and UV/O_3_ treatments. The study was then extended by the fabrication of recycled high-density polyethylene (rHDPE) composites reinforced with modified rice husk filler to achieve good mechanical properties. Lastly, the comparative study between the alkali, acid- and UV/O_3_-treated surface treatments on the biocomposites was analyzed.

## Experimental

### Raw materials

Rice husk (RH) which used as natural fiber filler was supplied by DinXings (M) Sdn Bhd with mesh size 212 μm while recycled high-density polyethylene (rHDPE) was used as a matrix in the biocomposites. The density of rHDPE is 923 kg/m^3^. The melt flow index (MFI) of rHDPE is 0.072 g/ 10 min at 190 ^o^C. The coupling agent used in this study was maleic anhydride grafted polyethylene (MAPE). Both rHDPE and MAPE were obtained from Muda Cemerlang Sdn. Bhd. RH was oven dried at 80°C for 24 h in order to reduce the moisture content and then stored in sealed plastic bag before mixing. Prior surface modification, RH was washed with distilled water to remove any impurities or dust on its surface.

### Surface treatment of rice husk

#### Alkali treatment

RH was soaked in a 0.5 M NaOH solutions for 2 h under stirring condition. The solution was then washed with distilled water again to remove the alkaline residues on RH surface until pH 7 shown. Lastly, treated RH was dried overnight at a temperature of 90°C using a vacuum oven. The parameter was adapted from Garcia et al. [28].

#### Acid treatment

Sulphuric and nitric acids at 3:1 (v/v) ratio were prepared to produce a high-concentration acid solution. Then, RH was soaked in the dilute acid solution and refluxed under the oxidation conditions. The oxidized RH was then filtered and washed with distilled water until they reached pH 7. Finally, the treated RH was dried in an oven at 90°C for 24 h.

#### Development of UV/O_3_ surface treatment

A self-made in-house UV/O_3_ system was used for this treatment. RH was placed inside the chamber and was exposed for 30 min exposure time and 10 L/min ozone flow rate. These parameters were followed based on the pilot experiments done in earlier studies [29]. In this study, the system was being fabricated internally by refering to United States Patent (Wydeven), Patent No: US 6,555,835 B1. The treatment process initiated by placing the sample in the conical flask which has ozone gas input and output flow channel and was placed in a reaction chamber with UV lamps (254 nm). Ozone generator has a function to generate ozone and channel it into the reaction chamber. The ozone gas that enters the chamber will react with UV rays where photosensitized oxidation process has taken place and forms carboxylic group at the surface of rice husk. In which photosensitized oxidation process is a process where the materials themselves are dissociated by the absorption of short-wavelength UV radiation [26].

### Fabrication of biocomposites

A laboratory scale counter-rotating twin screw extruder (Thermo Prism TSE 16PC) was employed for compounding RH fibers and rHDPE with the coupling agent in the first stage. Fiber loading of 30 wt% was used in this study. This was due to the 30 wt% of RH showed the optimum tensile properties of the composites produced based on our previous research [30]. The barrel temperatures of the four zones were 180 ^o^C, 190 ^o^C, 200 ^o^C and 190 ^o^C, and the screw speed were set at 30 rpm. After extrusion process, the extrudates were collected, cooled and granulated into pellets. The pellets collected were then pelletized using a crusher machine so that the pellets can fit into the mould. In the second stage, a compression moulding process was used to make the specimen panels for characterization using LP50, LABTECH Engineering Company LTD. Pellets were placed into a mould of 14 mm x 14 mm x 3 mm thick. The temperature of upper and lower platen of the hot press was set at 180°C. The period of preheating, venting and full pressing was set to 3, 2 and 5 min, respectively. Lastly, the cold press was set to 5 min to cool down the specimens. The pressure was maintained at 1000 psi to press the samples.

### Characterization

The infrared spectra in the Fourier Transform Infrared-Attenuated Total Reflectance (FTIR-ATR) of the raw and surface modified RH were analyzed using Perkin Elmer System 2000 FTIR Spectrophotometer in order to identify the changes in the chemical functional groups on the surface of the RH. Thermal stability of raw and unmodified RHs was studied using a Thermal Gravimetric Analyzer (TGA Q500 V20). The prepared composite specimens were characterized by tensile and notched impact tests. Tensile test was conducted using Materials Testing Machine, model: M350-10CT with a speed of 5 mm/min according to ASTM D 638–03. Ray-Ran Universal Pendulum Impact System was used to determine the impact strength of the specimens. The specimens were cut according to ASTM D 256–05 specification. The velocity of the pendulum was 3.46 m/s and the weight load used was 0.452 kg. The water absorption analysis was carried out based on the ASTM D 570–98 method. Three samples (76.2 × 25.4 × 3.0) mm for each formulation were dried in an oven for 24 h at 100°C to remove moisture. Each specimen was then weighed and immersed in distilled water at room temperature. The weight gains of specimen were determined periodically (2 h, 1 day, 7, 21, 35, 49 and 63 days). Specimens were then removed from the water, dried and measured. Water absorption (WA) of composites was computed using the following equation:

$$WA(\%)=\frac{W_{t}-W_{o}}{W_{o}}\times 100$$

where *Wo* is the weight of the dry specimen and *Wt* is the weight of the wet specimen at time *t*. Statistical comparisons on the measured data of mechanical, water absorption and wettability properties were performed using one-way analysis of variance (ANOVA) followed by post hoc t-test analysis with the aid of Data Analysis ToolPak in Ms Excel, to determine surface modifications effect at the 5% significance level (α = 0.05) and for combination between multiple groups. The micrographs of untreated and treated rice husks and the fractured surfaces of the composites were obtained using scanning electron microscope (SEM) (model: SUPRA 55VPSEM). The sample was sputter-coated with gold to make them conductive and was operated at an accelerating voltage of 15kV. The surface roughness and topography of the untreated and treated rice husk composites were determined by atomic force microscope (AFM) method using NT-MDT Model NTEGRA PRIMA and golden silicon cantilever (PROBE NSG01). Tapping mode AFM was conducted at a frequency of 1.0 Hz. Root-mean square (RMS) surface roughness of the 100 μm x100μm images was calculated using Image Analysis P9 software. Five scans were done at five different places on the same composite sample for untreated and treated RH composite. The wettability of the untreated and treated RH composites was determined by the contact angel measurement based on goniometry static water drop method using sessile drop techniques [31] using The Kruss Laboratory (DSA 100).

## Results and discussion

### Functional group analysis, thermal gravimetric analysis and morphology of various RH treatments

**Fig 1:**
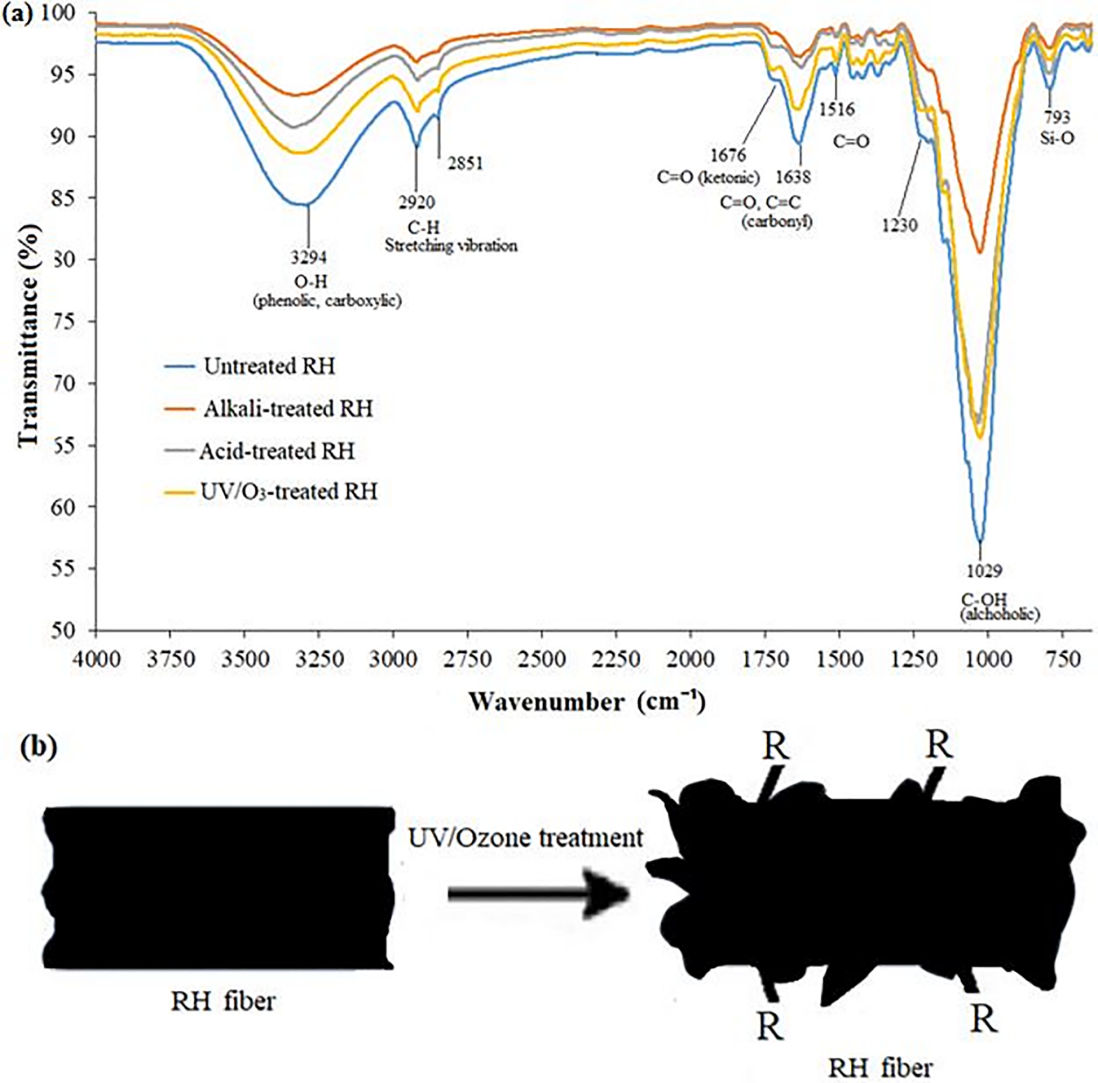
(a) FTIR spectra of untreated RH, alkali-treated RH, acid-treated RH and UV/O₃-treated RH, and (b) postulated chemical reaction of photosensitized oxidation process. R represents carboxyl, ester, carbonyl and hydroxyl functional groups.

Fig 1(A) shows the FTIR spectra of untreated RH and variuos surface modified-RH. For untreated RH, the spectrum shows a broad absorption band at approximately 3294 cm^-1^, which corresponding to the stretching vibration of hydroxyl (-OH) groups in the cellulose fibers [15]. Upon surface treatment, this absorption band has shifted to higher wavenumber, as 3310 cm^-1^ for alkali-treated RH, 3333 cm^-1^ for acid-treated RH and 3318 cm^-1^ for UV/O_3_-treated RH. This is an indication of the presence of free–OH groups in phenolic and carboxylic groups which do not take part in the formation of hydrogen bonding [32] during the treatment process. Besides, it is observed that there is a decrease in the intensity of these absorption peaks which possibly due to the chemical reaction of accessible–OH groups with the NaOH (alkali), H_2_SO_4_ and HNO_3_ (acid), and ozone (UV/O_3_)_._ The absorption vibration that is appeared in 1638–1676 cm^-1^ for untreated RH represents to the vibration of carbonyl from carboxylic groups in ester linkages which are attributed to the wax and natural fats, as proposed by Trejo-O'reilly [33]. The peak intensity at this absorption vibration range is found to decrease upon the treatments, which indicates the removal of wax and natural fats. The O-Si-O stretching vibrations that are obtained at 1033 cm^-1^ and 793 cm^-1^ corresponds to the silica content in rice husk, thereby the removal of silica upon surface treatment and the degradation of a part of the cellulose embedded with silica on the rice husks during disintegration of this membrane causes the intensity of these absorption peaks to be lowered or the disappearance of peaks. Comparing to untreated and other treated RH, UV/O_3_-treated RH shows the obvious peaks at 1725 cm^-1^ and 1641 cm^-1^ which are associated to the ketonic stretching and carbonyl group or quinone, respectively [34]. Ozone exists as both molecular and atomic oxygen on the surface of carbon where atomic oxygen is a powerful oxidizing agent. Hence, upon ozone treatment, the atomic oxygen oxidizes the carbon surface into functional groups such as carboxylic, ketonic and phenolic [34]. Meanwhile, the carbon atom that is either owing to the UV radiation or originally existing at the defect sites on the RH tends to react with the atomic oxygen from the continuous dissociations of the oxygen molecules [26]. Fig 1(B) below shows the formation of ozone molecules for the photosensitized reaction. The chemical functional group present is denoted by ‘R’, where it represents all the groups present after the treatment i.e. carboxyl, ester, carbonyl and hydroxyl groups [34]. All these peaks indicate a better adhesive surface is formed on treated RH, and thus improving the bonding between the polymer matrix and the RH filler for the fabricated composite.

**Fig 2:**
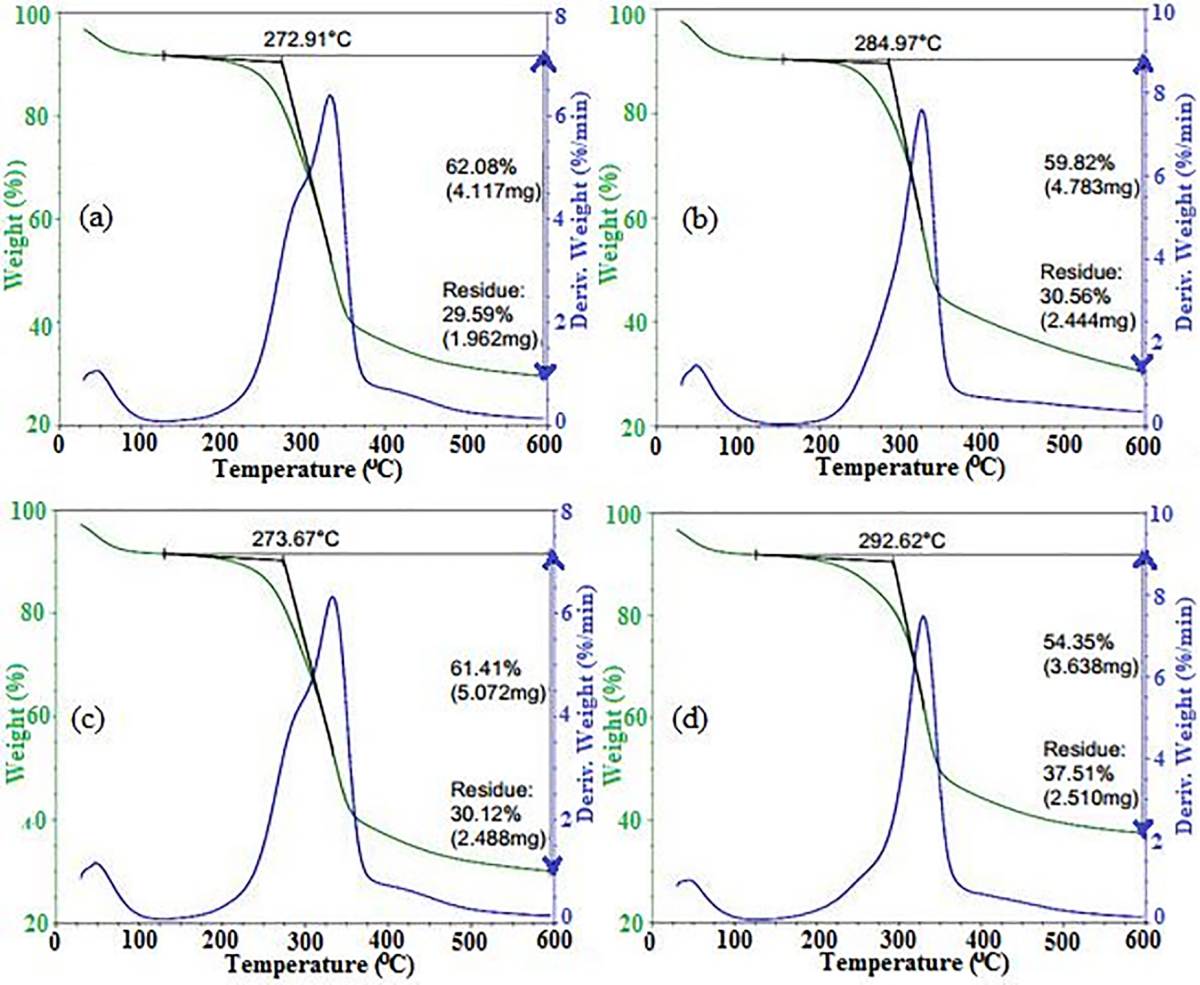
TGA degradation curve of (a) untreated RH, (b) alkali-treated RH, (c) acid-treated RH and (d) UV/O₃-treated RH.

The thermal stability of the untreated RH and surface modified RH was investigated and the results are shown in Fig 2. All the raw and treated RH undergo initial weight loss between 60–200°C, irrespective of their treatment condition. This weight loss is due to the removal of water and other primary volatile substances [35]. Most of the thermal degradation take place in between 260°C– 360°C where this implies the decomposition of secondary volatile substances such as primary hemicelluloses and cellulose [36]. In comparison to untreated RH (272.91°C), the degradation of surface modified RH occurs at higher temperature as 284.97°C and 292.62°C for the alkali-treated, and UV/O_3_-treated RH, respectively, except for the insignificant enhancement in the acid-treated RH (273.67°C), as shown by the sharp peaks as shown on the derivative thermal gravimetric (DTG) curve. This indicates the enhanced thermal stability of RH upon the alkali and UV/O_3_ treatments. Interestingly, the decomposition of alkali-treated and UV/O_3_-treated RH involves only one step process, in contrast to those of untreated RH and acid-treated RH which exhibit a sharp peak at 335–340°C (decomposition of cellulose component) with a shoulder peak at 230–310°C (depolymerization of hemicellulose component) [37]. The disappearance of the shoulder step confirms the removal of hemicelluloses and wax during the surface treatment. As reported by Gassan et al. [20], the chemical constituents of natural fiber like cellulose, hemicellulose and lignin are very sensitive UV radiation in which a deterioration effect could occur more rapidly by the presence of UV light. However, lignin which acts as the surface constituent of a natural fiber has a strong UV-absorbing characteristic and hence, it may provide the shielding effect to protect cellulosic components from excessive photo-degradation as well as thermal degradation.

**Fig 3:**
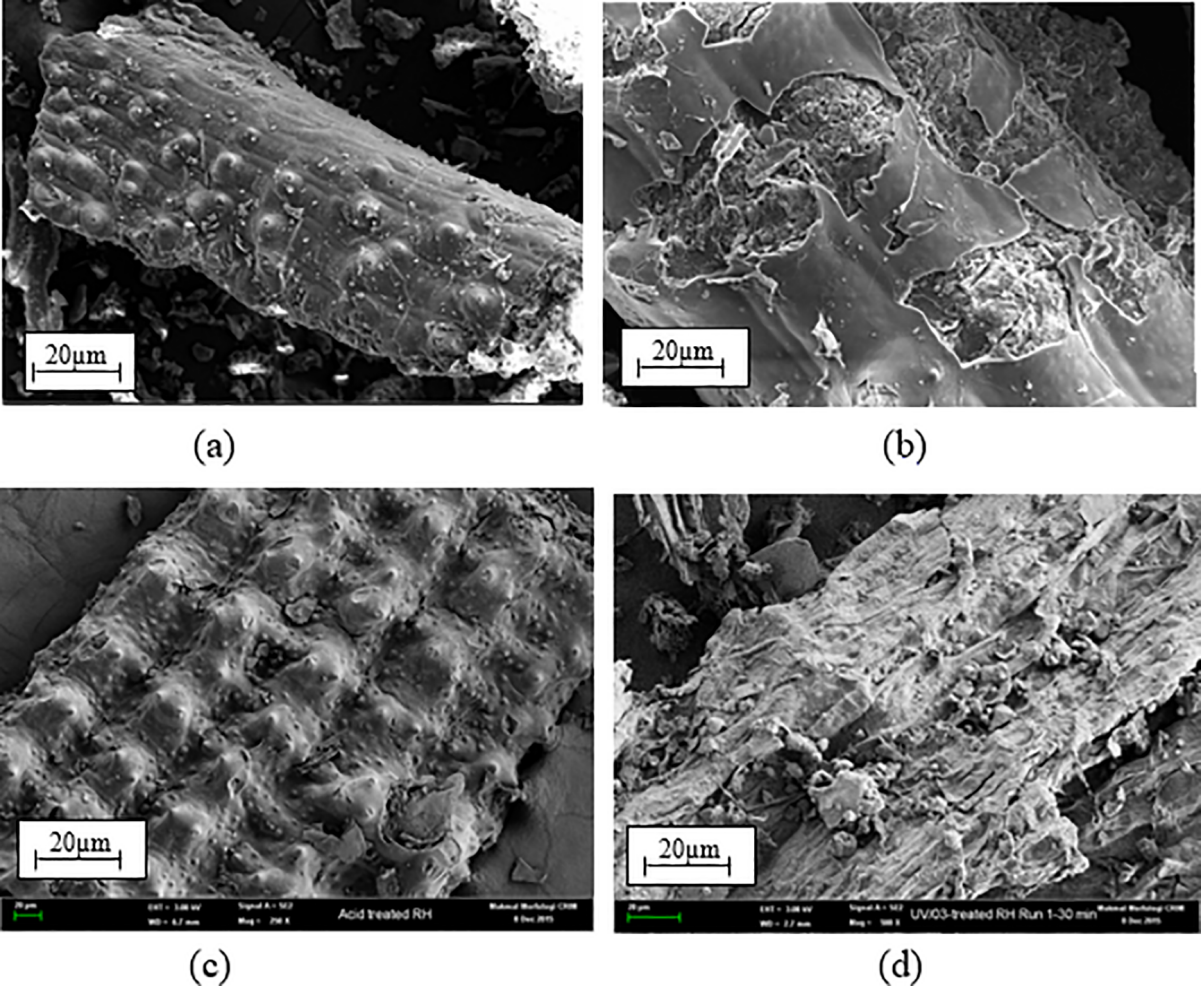
SEM morphology of (a) untreated RH, (b) alkali-treated RH, (c) acid-treated RH and (d) UV/O₃-treated RH.

The SEM micrographs of the surfaces of untreated RH and treated RH are shown in Fig 3. The results show that the surface of untreated-RH (Fig 3(A)) possesses a smooth, cloudy and flat surface. This is due to the existence of wax, lignin and hemicelluloses in raw RH constituents [38]. Upon alkaline treatment, the lignin, hemicelluloses and other impurities dissolve in alkaline solution, subsequently are being removed from the surface of alkali-treated RH. Therefore, the alkali-treated RH possesses a rougher surface, as shown in Fig 3(B). This observation has been reported by Chen and co-researchers [12] who carried out alkali treatment on RH. On the other hands, acid-treated RH and UV/O_3_-treated RH also show rough surfaces which indicating the surfaces had been treated. A rougher surface promotes the mechanical interlocking and enhancement in the compatibility between fibres and matrix [38].

### Mechanical, physical and surface properties of untreated and surface modified RH biocomposites

#### Mechanical performance

**Fig 4:**
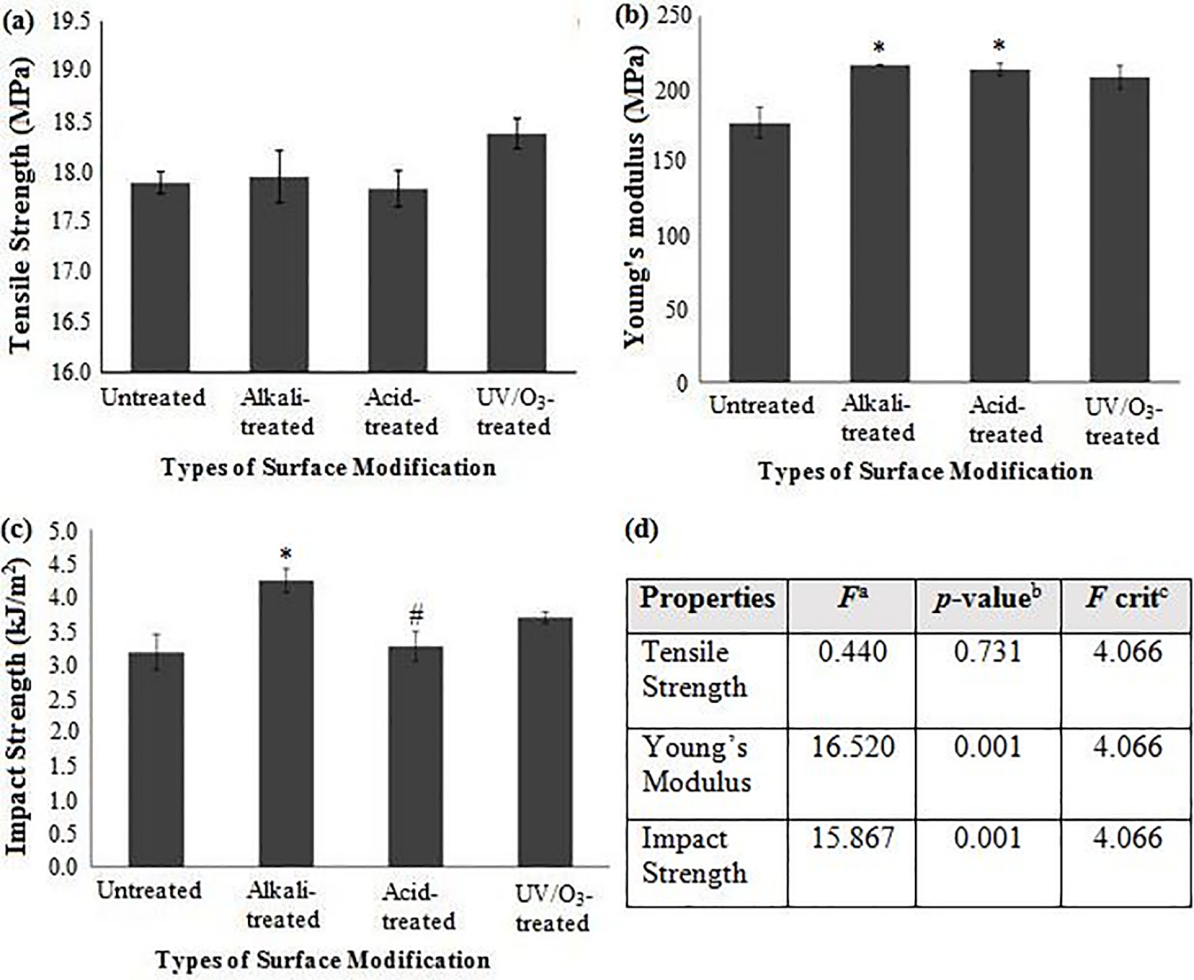
(a) Tensile strength, (b) Young’s Modulus, and (c) Impact strength of composites with 30wt% untreated, alkali-treated, acid-treated and UV/O3-treated RH composites; (d) ANOVA of mechanical properties of RH/rHDPE composites. Note: ^a^*F* ratio = mean between groups variance/mean within group variance; ^b^Probability from 0 to 1; ^c^critical *F*-value based on F distribution. Groups with a significant difference (p < 0.05) compared to untreated RH composite and alkali-treated RH composite are indicated by * and # symbols, respectively.

Tensile test has been performed to measure the response of a material to slowly applied uniaxial force [39]. Fig 4(A) and Fig 4(B) illustrate the tensile strength and tensile modulus of composites with 30 wt% of RH of untreated, alkali-treated, acid-treated and UV/O_3_-treated RH, respectively. Upon alkali and acid treatments, the tensile strengths of composites containing 30 wt% RH show no statistical differences (*p*-value = 0.731, > 0.05; *F*-values < F-critical values) between control (untreated) and any treated groups, as displayed in Fig 4(A). In the case of alkali treatment, the tensile strength result in this current study is not in agreement with the previous finding where the treated-hemp fiber at 40% (volume fraction) reinforced polylactic acid composites has increased the tensile strength up to 18% as compared to the untreated fiber composites [13]. The possible interpretations are the different polarity of the polymer matrix where the HDPE is hydrophobic and PLA is hydrophilic so the same treatment does not confirm the effectiveness for all cases; and there is another surface modifying agent (MAPE) used in this current study. It can be seen that UV/O_3_-treated RH exhibits the highest tensile strength as compared to the untreated and other RH treatments. The possible reason for this trend is that the physical and chemical degradation that are taken place in alkali and acid treated RH have led to significant degradation in particles rigidity [12, 38], in which these types of degradation do not occur for milder treatment, for instance, using UV/O₃. It can be concluded that UV/O_3_ treatment shows an optimum result and can be used as an alternative surface modification method in RH composites. Nevertheless, the post hoc t-test analysis shows insignificance among the treated groups.

In Fig 4(B), Young’s Modulus of surface modified RH composites shows higher values than that of untreated RH composites, with *p*-value between groups at 0.001 (<0.05) and *F* values are greater than F-critical. The post hoc test shows a significance exists between the control and alkali-treated group as well as between the control and acid-treated composites as shown by the marked “*” symbol in Fig 4(B). This significant improvement is believed to be due to the increase of stiffness of RH after treatment. The treated RH fibres tend to remove the lignin, wax, and other impurities from the external surface of the fibre which make the surface of the fibre rougher by revealing the fibrils and more accessible OH groups, thereby giving a good mechanical interlocking between the fibres and matrix polymers [40]. However, among the surface modifications, the Young’s Modulus does not show significant differences. This is attributed to which Young’s Modulus is believed to be more correlated with the intrinsic characteristics of the composite materials and the stiffness of this composite is imparted by RH fillers [41]. In this case, the amount of RH filler added into the polymer matrix is the same. Therefore, the obtained results of Young’s Modulus is logical.

Impact test has been conducted to measure the ability of a material to absorb the sudden application of a load with breaking. Fig 4(C) shows the results of the impact strength of composites with 30 wt% of untreated, alkali-treated, acid-treated and UV/O_3_-treated RH. The impact strength of the untreated RH composites shows a relatively low result due to the poor adhesion bonding between the filler and the polymer matrix [42]. The ANOVA results (Fig 4(D)) proved that there are statistically significant differences in the impact strength versus different treatments, *p*-value between groups is less than 0.05 (0.001) and *F* values are greater than F-critical (15.867 > 4.066). A significant improvement in the impact strength is observed for alkali-treated composite compared to untreated composite, as shown by the marked “*” symbol. Another significance exists between alkali- and acid-treated composite groups, as indicated by “#’ symbol. Upon alkali treatment, the removal of surface impurities on RH surface seems favourable in improving the filler-matrix adhesion and thus increasing the composite toughness. In another word, there exists a higher force transfer potential at the fibre-matrix interface in the composites with alkali-treated fibres. However, acid treatment only shows a slight improvement (with about 3% increment) on its impact strength compared to the untreated RH. The statistical post hoc t-test analysis supports this insignificant trend. As reported earlier, acid acts as one of the most effective reagents for the structural breakdown which includes the removal of lignin and hemicellulose, the reduction of cellulose crystallinity and the increase of porosity [11], hence acid treatment on RH surface obstructs the improvement in impact strength of RH composites.

#### Water absorption behaviour in distilled water

**Fig 5:**
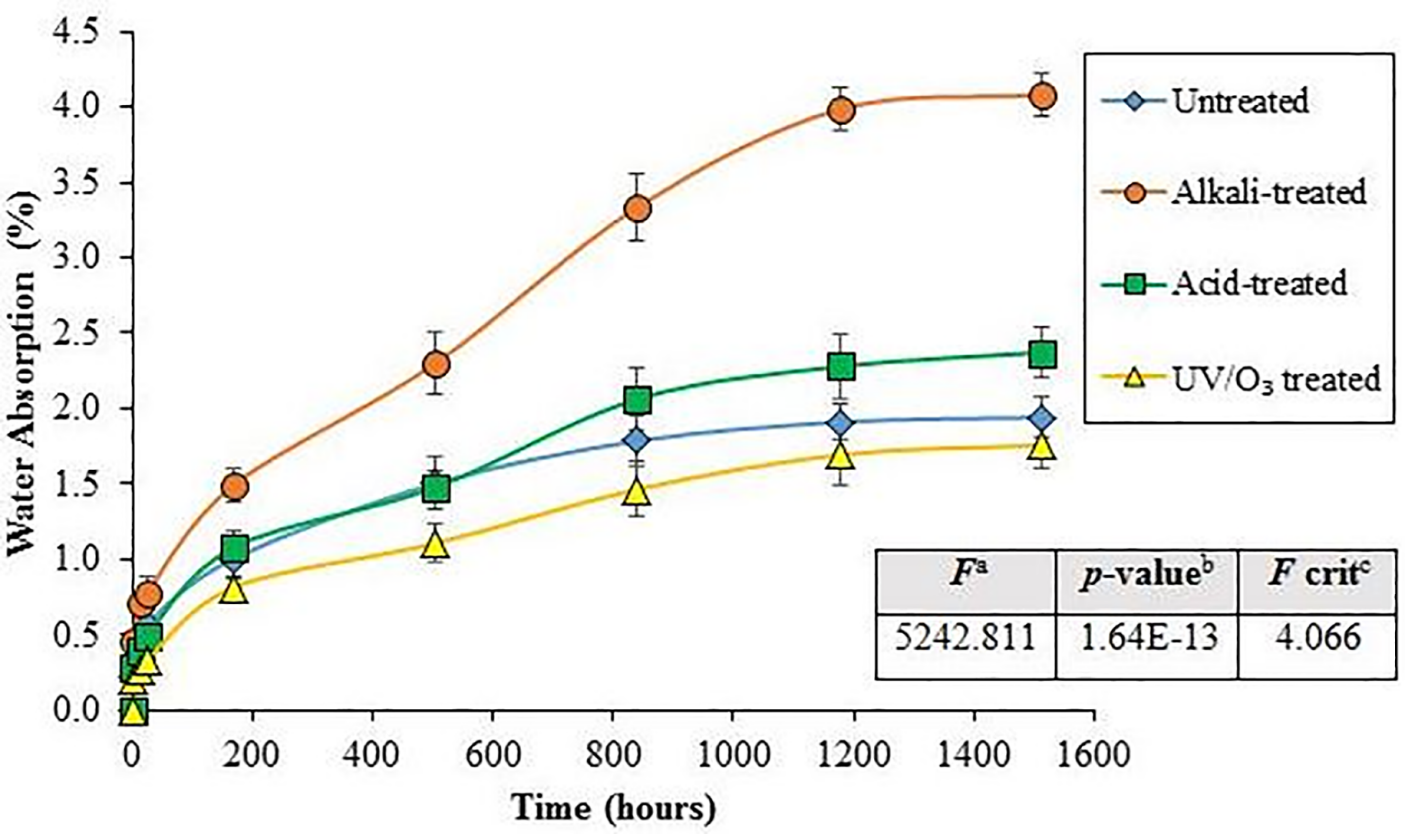
Water absorption of composites with 30wt% various surface-treated RH. The inset table shows ANOVA results. Note: ^a^*F* ratio = mean between groups variance/ mean within group variance; ^b^Probability from 0 to 1; ^c^critical *F*-value based on F distribution.

Water absorption in natural fiber/polymer composites are generally induced by two possible mechanisms. Firstly, the direct diffusion of water molecules through the micro gaps between polymer chains and secondly, the direct diffusion of water molecules through capillary transport into the gaps and flaws at the interfaces between the fiber and matrix [43]. The percentages of water absorption for untreated RH/rHDPE composite, alkali-treated RH/rHDPE composite, acid-treated RH/rHDPE composite and UV/O_3_-treated RH/rHDPE composite are demonstrated in Fig 5. The water absorption at saturation point (*M*_*m*_) and the values of diffusion kinetic parameters *n* and *k* that determined from the Fick’s equation [44] are tabulated in Table 1. All the composite formulations exhibit Fickian behaviour regardless of type of treatment, as evident from the values of *n* which are close to 0.5. Therefore, the results obey Ficks’ water absorption model [45, 46].

**Table 1:** Water absorption at saturation point (*M*_*m*_) and values of diffusion parameters *n* and *k*.

RH/rHDPE biocomposites with various surface modifications	*M*_*m*_ *(%)*	*n*	*k* (hour^-n^)
Untreated	1.942 ± 0.132	0.528	0.040
Alkali-treated	4.082 ± 0.145*	0.523	0.036
Acid-treated	2.371 ± 0.155*^#^	0.504	0.031
UV/O_3_-treated	1.758 ± 0.165*^#♦^	0.528	0.023

*M*_*m*_ value for each sample is the mean ± standard deviation.

Groups with a significant difference (p < 0.05) compared to untreated RH composite, alkali-treated RH composite and acid-treated RH composite are indicated by *, # and ♦ symbols, respectively.

As listed in Table 1, the *M*_*m*_ is found to achieved at 1.942% (untreated), 4.082% (alkali-treated), 2.371% (acid-treated) and 1.758% (UV/O_3_), respectively. Comparing to the control (untreated) group, the results presented here demonstrate that the surface modification on RH fibers causes a significant effect (p<0.05; differences between the control and treated groups) on the water absorption in equilibrium state of composites. It is found that the alkali-treated RH/rHDPE composites exhibit the highest water absorption. This can be attributed to the expansion of a specific surface area of the filler due to the fibre swelling after the process of alkaline immersion (mercerisation). This phenomenon has increased the accessibility of water molecules in the reactive region. As a result, the hydrophilic nature of the filler increases [47]. Therefore, alkali-treated composites exhibit the highest water absorption among the untreated (shown by “*” symbol marked at the alkali-treated bar in Fig 5) and other composites containing RH treated by acids and UV/O_3_ treatment (a significance exists between alkali-treated and acid-treated groups as well as between alkali-treated and UV/O_3_-treated groups, respectively, as indicated by “#” symbol). Comparing to untreated RH composite, the acid-treated RH composite possesses a higher water absorption due to the increased hydrophilic nature of RH but it is not to the extent of alkali treatment. Interestingly, the UV/O_3_-treated RH/rHDPE composites exhibit even lower water absorption as compared to the untreated RH composites. This may be ascribed to the dry treatment of UV/O_3_ where the RH was not soaked in any liquid medium during the treatment. Comparing to alkali and acid treatments, the expansion of specific surface area of the UV/O_3_-treated fibre can be avoid and thus, the water molecules are harder to diffuse into the fibres. In which the lower specific surface area of the UV/O_3_-treated RH fibre can be confirmed by its lower surface thickness compared to the acid- and alkali-treated RH as obtained in AFM result (will be discussed later). Therefore, the result clearly shows that UV/O_3_ surface treatment exhibits better resistance of water absorption as compared to all other types of surface treatments.

#### Morphological observation

**Fig 6:**
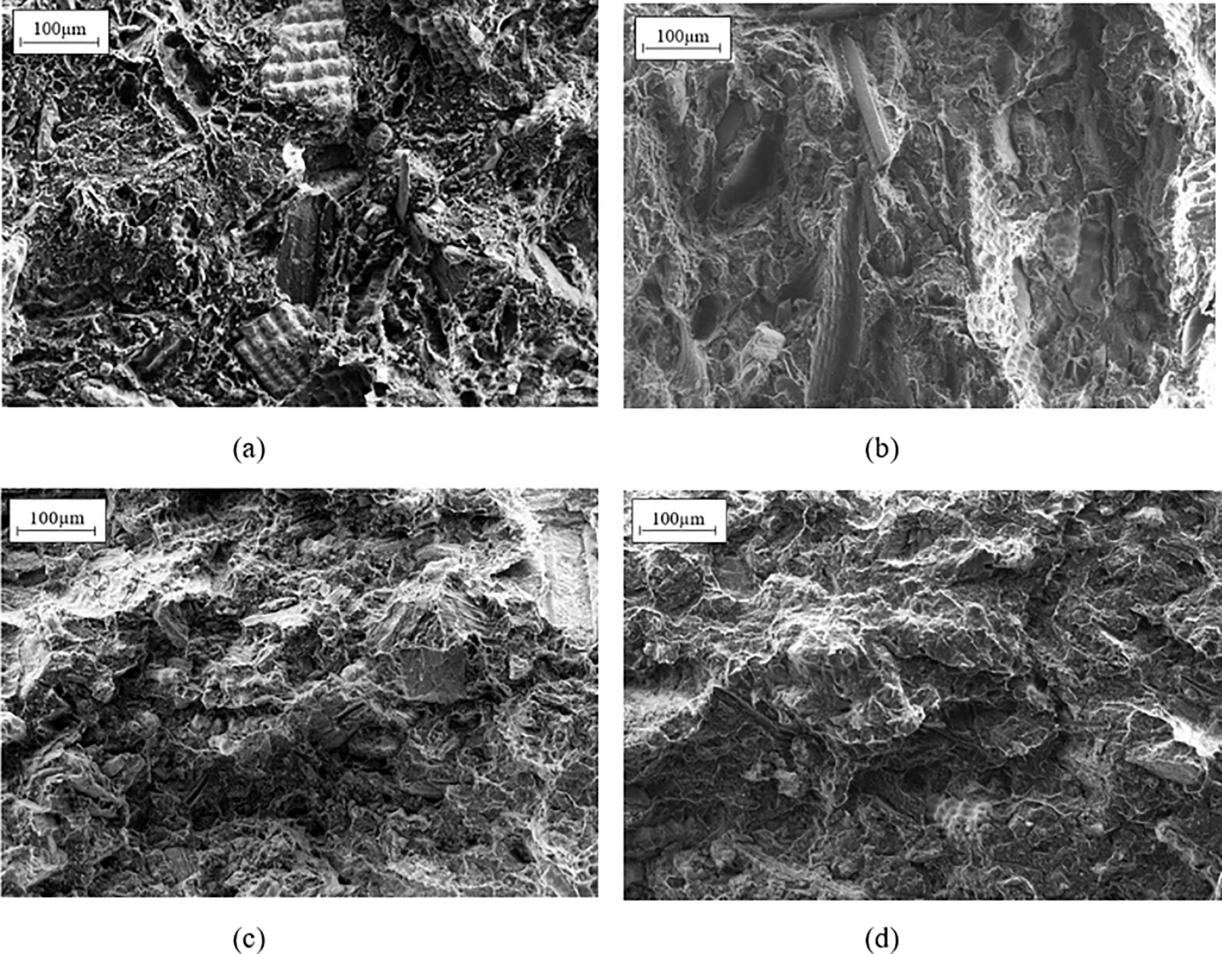
SEM Morphology of composites with 30wt% RH loading of (a) untreated, (b) alkali-treated, (c) acid-treated and (d) UV/O_3_-treated RH.

(Fig 6A–6D) show the SEM micrographs of untreated, alkali-treated, acid-treated and UV/O_3_-treated RH composites using magnifications of 50x. From the Fig 6(A), we can obviously see that generally the morphology structure of untreated RH composites is loosely attached in which there are numbers of isolated RH flakes and more cavities resulted from the fiber pullouts. This observation implies that the is a lack of strong physical interaction and adhesion between the untreated RH filler and polymer matrix. Conversely, the surface-treated RH is found to embed into the polymer matrix as a result of the improved interfacial adhesion and linking. Due to the filler-matrix interaction, it is visible that less cavities exist in the morphologies of treated composites as compared that of to the untreated composites. The similar observations have been reported in the previous research works on rice husk reinforced polyethylene [48] and natural rubber/HDPE composite [40]. In short words, all surface modifications promote a more adhesive surface of treated RH to adhere with the polymer matrix during composite processing.

**Fig 7:**
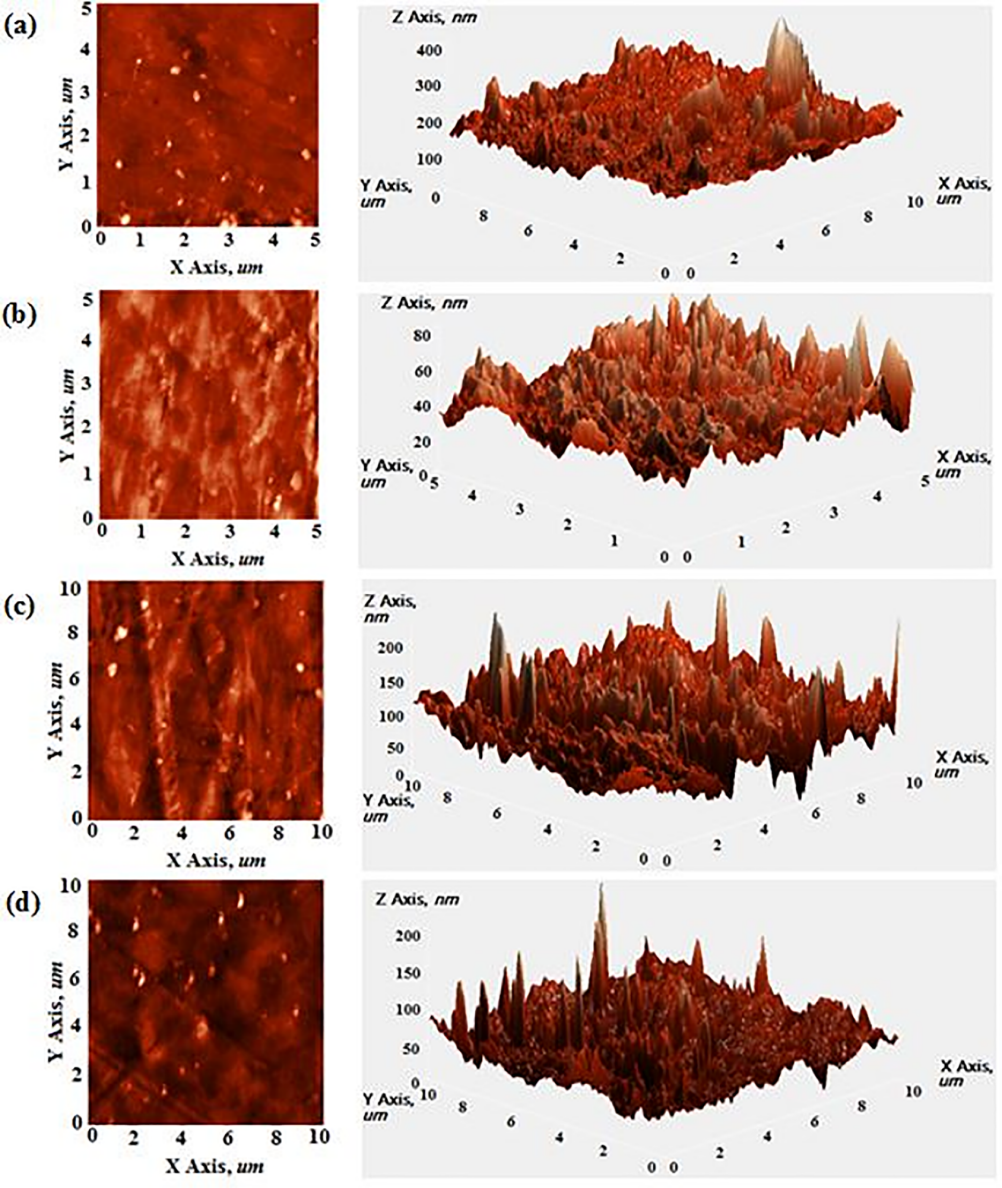
Atomic force microscope images of (a) untreated RH/rHDPE composite with RMS roughness = 10.25 ± 0.73 nm, (b) alkali-treated RH/rHDPE composite with RMS roughness = 18.46 ± 0.43 nm, (c) acid-treated RH/rHDPE composite with RMS roughness = 27.58 ± 0.76 nm and (d) UV/O_3_-treated RH/rHDPE composite, RMS roughness = 15.45 ± 0.51 nm, height view (left) and their respective 3D view (right).

Atomic force microscopy (AFM) has been used to characterize the changes in the surface topography and morphology of the samples resulting from surface modification [49]. Fig 7 signifies the topographical images of untreated, alkali-treated, acid-treated and UV/O_3_-treated RH/rHDPE composites in their height view (left) and 3D view (right). The surface roughness (RMS roughness) of untreated RH/rHDPE composite is 10.25 **±** 0.73 nm. Upon surface treatment, there is an increase in the RMS roughness for all surface treatment by about 50–169%, due to the eruption during the treatment processes [50]. This justifies that surface modification promotes rougher surface for better mechanical interlocking as discussed in SEM earlier.

### Wettability analysis

**Fig 8:**
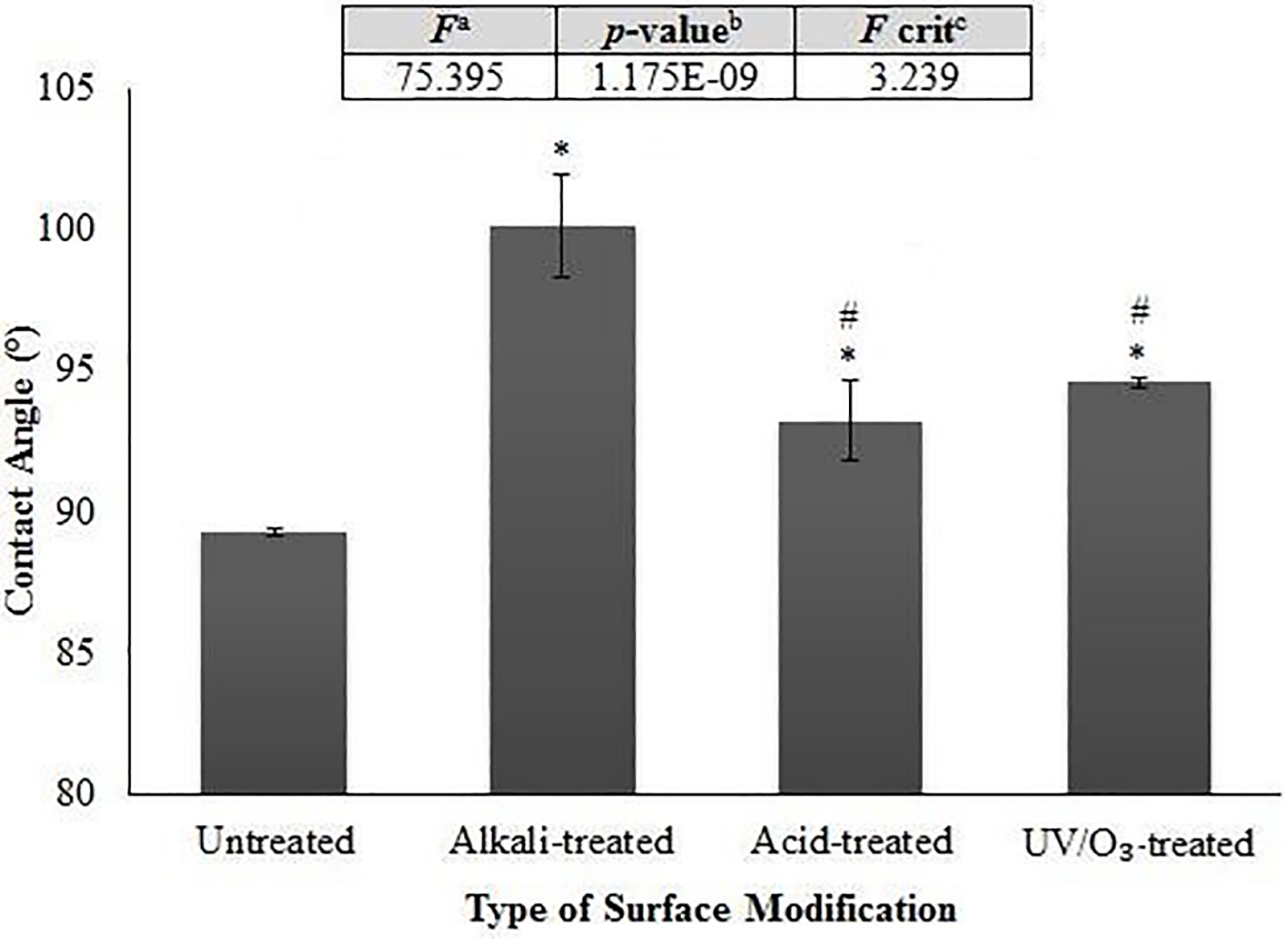
Contact angle of composites with 30wt% RH loading of (a) untreated, (b) alkali-treated, (c) acid-treated, and (d) UV/O3-treated RH. Note: ^a^*F* ratio = mean between groups variance/mean within group variance; ^b^Probability from 0 to 1; ^c^critical *F*-value based on F distribution. Groups with a significant difference (p < 0.05) compared to untreated RH composite and alkali-treated RH composite are indicated by * and # symbols, respectively.

Wettability analysis was conducted by the common contact angle measurement. Contact angles give a valuable information about the degree to which liquids wet a fiber. It also determines how easy is the liquid to penetrate the fiber accumulations [51]. Fig 8 shows the contact angle of RH composites under the investigated treatments. It can be seen that all surface modifications improve the wettability of the RH composites in which the degrees of contact angle are higher for modified RH-composites (93.5–100°) as compared to the untreated ones (89.3°). This result is in agreement with the research carried out by Švorčík, Makajová [52]. A significance exists between the control and alkali-treated group, between the control and acid-treated group, and between the control and UVO_3_-treated group, respectively, as shown via post hoc t-test analysis after ANOVA. Upon treatment, lignin has been removed thus making the rice husk filler to be more hydrophobic and higher resistance to water. Among all the surface modifications, alkali-treated RH composites show the highest wettability with the highest contact angle. This trend is supported by the FTIR results which show the lowest intensity of absorption peak at 3310 cm^-1^ (corresponding to the hydroxyl group).

## Conclusions

This study investigated the effect of modification of rice husk (RH) surfaces by alkali, acid and ultraviolet-ozonolysis (UV/O_3_) treatment on the properties of RH/rHDPE composites. Upon surface modification on RH, the RH fiber displayed a rougher surface than before treatment. The treated RH possessed the improved thermal stabilities, especially for alkaline and UV/O_3_ treatment. Tensile test showed that UV/O_3_-treated RH gave the maximum tensile strength in composites compared to the untreated and other RH treatments. Impact results showed a significant improvement upon alkali and UV/O_3_ treatment, whereas acid treatment only showed slight improvement. Therefore, it can be concluded that UV/O_3_ treatment showed an optimum result and can be used as an alternative surface modification method in RH composites in order to enhance the mechanical properties of composites.
